# Recommendation Reversals in Gastroenterology Clinical Practice Guidelines

**DOI:** 10.1093/jcag/gwab040

**Published:** 2021-10-21

**Authors:** Reza Gholami, Rishad Khan, Anushka Ramkissoon, Abdulrahman Alabdulqader, Nikko Gimpaya, Rishi Bansal, Michael A Scaffidi, Vinay Prasad, Allan S Detsky, Jeffrey P Baker, Samir C Grover

**Affiliations:** Division of Gastroenterology, St. Michael’s Hospital, Toronto, Ontario, Canada; Department of Medicine, University of Toronto, Toronto, Ontario, Canada; Division of Gastroenterology, St. Michael’s Hospital, Toronto, Ontario, Canada; Division of Gastroenterology, St. Michael’s Hospital, Toronto, Ontario, Canada; Division of Gastroenterology, St. Michael’s Hospital, Toronto, Ontario, Canada; Division of Gastroenterology, St. Michael’s Hospital, Toronto, Ontario, Canada; Division of Gastroenterology, St. Michael’s Hospital, Toronto, Ontario, Canada; Department of Epidemiology and Biostatistics, University of California San Francisco, San Francisco, California,USA; Institute of Health Policy, Management and Evaluation, Dalla Lana School of Public Health, University of Toronto, Toronto, Ontario, Canada; Department of Medicine, Mount Sinai Hospital and University Health Network, Toronto, Ontario, Canada; Division of Gastroenterology, St. Michael’s Hospital, Toronto, Ontario, Canada; Department of Medicine, University of Toronto, Toronto, Ontario, Canada; Division of Gastroenterology, St. Michael’s Hospital, Toronto, Ontario, Canada; Department of Medicine, University of Toronto, Toronto, Ontario, Canada; Li Ka Shing Knowledge Institute, St. Michael’s Hospital, Toronto, Ontario, Canada

**Keywords:** Clinical practice guidelines, Evidence-based medicine, Quality of care

## Abstract

**Background:**

Recommendations in clinical practice guidelines (CPGs) may be reversed when evidence emerges to show they are futile or unsafe. In this study, we identified and characterized recommendation reversals in gastroenterology CPGs.

**Methods:**

We searched CPGs published by 20 gastroenterology societies from January 1990 to December 2019. We included guidelines which had at least two iterations of the same topic. We defined reversals as when (a) the more recent iteration of a CPG recommends against a specific practice that was previously recommend in an earlier iteration of a CPG from the same body, and (b) the recommendation in the previous iteration of the CPG is not replaced by a new diagnostic or therapeutic recommendation in the more recent iteration of the CPG. The primary outcome was the number of recommendation reversals. Secondary outcomes included the strength of recommendations and quality of evidence cited for reversals.

**Results:**

Twenty societies published 1022 CPGs from 1990 to 2019. Our sample for analysis included 129 unique CPGs. There were 11 recommendation reversals from 10 guidelines. New evidence was presented for 10 recommendation reversals. Meta-analyses were cited for two reversals, and randomized controlled trials (RCTs) for seven reversals. Recommendations were stronger after the reversal for three cases, weaker in two cases, and of similar strength in three cases. We were unable to compare recommendation strengths for three reversals.

**Conclusion:**

Recommendation reversals in gastroenterology CPGs are uncommon but highlight low value or harmful practices.

**SUMMARY BOX**

**What is already known about this subject?**

Recommendations in gastroenterology practice guidelines are often based on low quality evidence. Recommendations may be reversed when new evidence shows they are unsafe or futile.

**What are the new findings?**

We identified 11 recommendation reversals among 129 guidelines from 20 subspecialty societies spanning a range of topics in clinical gastroenterology. While rare, recommendation reversals highlight low-value clinical practices that may waste resources and cause harm to patients.

**How might this impact clinical practice in the foreseeable future?**

Future trials, and a robust agenda, should assess whether longstanding dogma or recommendations in gastroenterology truly improve patient outcomes. In order to deliver the best medical care to our patients, revising recommendations in CPGs based on new evidence is paramount.

## Background

Recommendations in clinical practice guidelines (CPGs) should be updated as new evidence emerges (1–3). While this evidence often leads to the inclusion of new therapies in guidelines, recommendations may also be reversed when they are shown to be futile or harmful (4,5). Identifying these cases of recommendation reversal is of key importance and should purge practices that jeopardize patient safety and healthcare resource management.

Practices in clinical gastroenterology change over time. For example, thiopurine therapy was believed to be potentially beneficial in early Crohn’s disease (6). The *Azathioprine for Treatment of Early Crohn’s Disease in Adults* (AZTEC) trial (7), however, found it to have no effect on clinical remission and put patients at increased risk of adverse events when compared with placebo. When novel medical evidence leads to the removal of a prior recommendation, we refer to the phenomenon as a *recommendation reversal*. In this instance, despite the small sample size and wide confidence interval for the primary outcome, the AZTEC trial led the European Crohn’s and Colitis Organization (ECCO) to recommend against early introduction with thiopurines in newly diagnosed Crohn’s disease (8).

Many recommendations in gastroenterology society guidelines are based on weak evidence (9–11). Across medical specialties, practices that are promoted based on poor quality data or pathophysiological rationales can be subsequently found to be futile or harmful (12,13). Well-known examples include stenting for stable coronary artery disease and routine pulmonary artery catheterization for critically ill patients (14–16). Prior work has examined specific medical practices across fields, but the frequency of recommendation reversals in clinical practice guidelines is unknown. For this reason, we identified and characterized recommendation reversals among major gastroenterology society CPGs.

## METHODS

We systematically searched all clinical practice guidelines published by select gastroenterology societies from January 1990 to December 2019 and identified recommendation reversals as defined below.

### Clinical Practice Guideline Identification

We included North American, European, Asian, Australian, and international societies that produced guidelines in English primarily for topics in gastroenterology, including endoscopy, hepatology, inflammatory bowel disease, nutrition, and gastrointestinal surgery. We did not include societies that publish guidelines on topics that include illnesses that affect the gastrointestinal system such as oncology or primary care. Supplementary Table 1 lists the societies included in this study.

Two authors (R.G. and A.R.) hand-searched, independently and in duplicate, all CPGs published by these societies during the period of January 1990 to December 2019. The CPGs were collected from the societies’ respective websites. As we were interested in recommendations that changes over time, we only included guidelines that had two or more iterations on the same topic. We included guidelines that graded the strength of their recommendations and/or evidence. We excluded position statements and clinical pathway documents that were not based on evidence.

### Data Extraction

The two authors used a standardized data collection form to record the following information: name of society producing the guideline, guideline topic, iteration, year of publication, and type of evidence grading system. Explicit recommendations were extracted and categorized based on topic, and strength of recommendation and level of evidence as rated by the guideline authors/committee members. We compared recommendations addressing the same clinical practice in different guideline iterations from the same society. We then identified all instances of recommendation reversals. Evidence cited in both the original and reversed recommendations were recorded. Discrepancies with respect to data collection and recommendation reversal inclusion were resolved by a third author (R.K.).

We defined a recommendation reversal using two criteria, both of which were required for inclusion:

a. The most recent iteration of a CPG recommended against a specific practice, in contradiction of a previous iteration of the CPG from the same body that recommended the opposite.
b. The recommendation in the previous iteration of the CPG is not replaced by a new diagnostic or therapeutic recommendation in the more recent iteration of the CPG.

Two other authors (N.G., S.C.G.) reviewed all identified reversals to ensure that the above two criteria were met. For each reversal that was identified, we also noted both the strength of recommendations and the quality of the underlying evidence for the newer recommendation.

### Outcomes and Data Analysis

The primary outcome was the number of recommendation reversals. Secondary outcomes included new evidence provided in CPGs for recommendation reversals, and changes in recommendation strength after a recommendation was reversed. All data retrieved from the CPGs were managed using Microsoft Excel (2019).

## RESULTS

**Figure 1.**
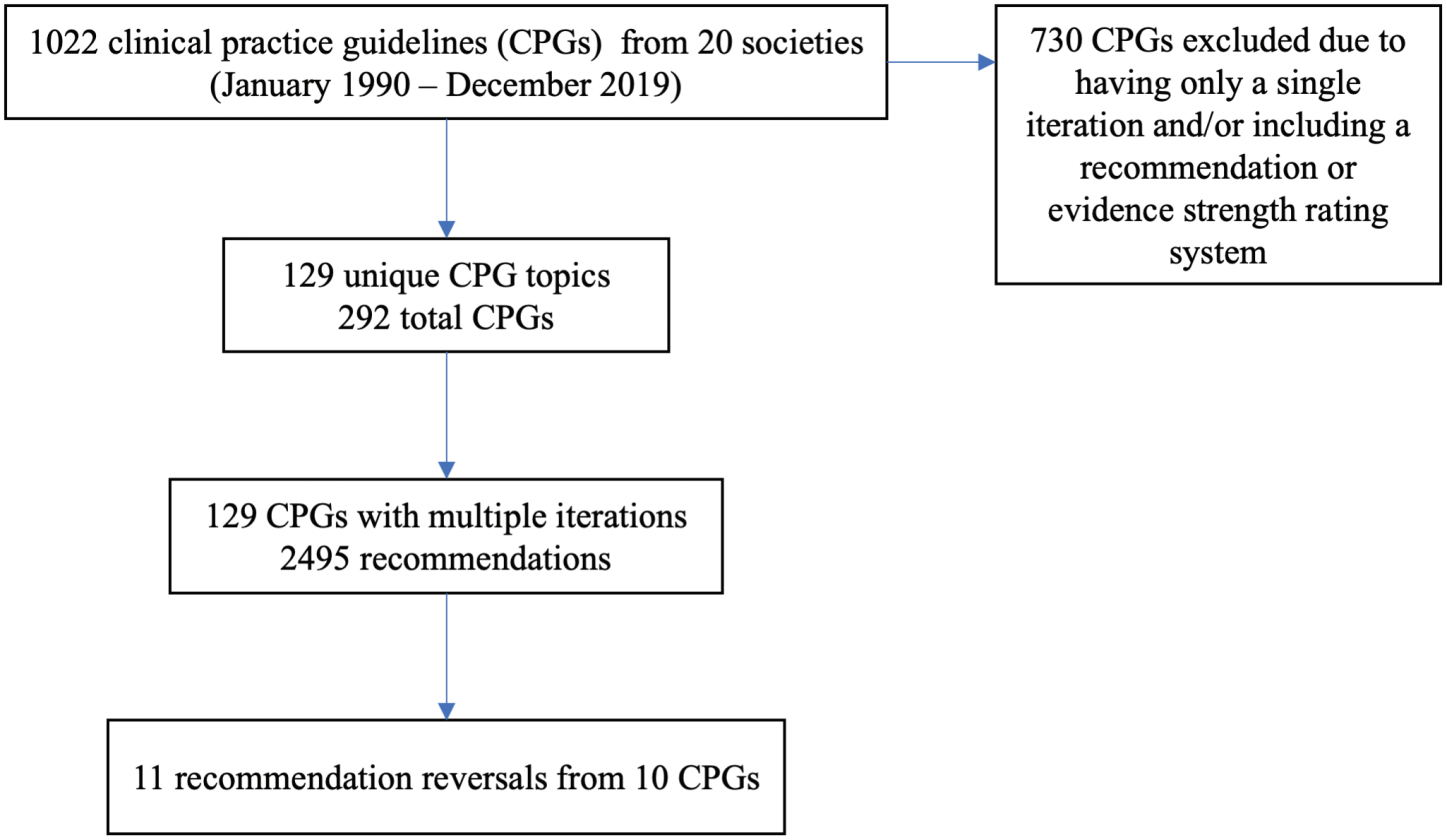
Study flow diagram.

Twenty societies published a total of 1022 CPGs from 1990 to 2019. There were 129 unique guideline topics for which there was more than one iteration. Our final sample consisted of 129 final iterations of CPGs, and 292 total CPGs when considering each iteration as an individual guideline (Figure 1). Guidelines were from a range of gastroenterology societies from North America, Europe, and Asia (Table 1).

**Table 1.** Final sample of clinical practice guidelines

Society	Number of guidelines (*N* = 129)	Number of reversals (*N* = 11)
American Association for the Study of Liver Diseases	13	2
American College of Gastroenterology	6	1
American Gastroenterological Association	3	0
American Society of Colon and Rectal Surgeons	21	1
American Society for Gastrointestinal Endoscopy	29	2
American Society for Parenteral and Enteral Nutrition	1	0
British Society of Gastroenterology	12	1
Canadian Association of Gastroenterology	3	0
European Association for the Study of the Liver	5	1
European Crohn’s and Colitis Organization	6	1
European Society for Clinical Nutrition and Metabolism	16	0
European Society of Gastrointestinal Endoscopy	10	1
Japan Gastroenterological Endoscopy Society	1	1
Society of American Gastrointestinal and Endoscopic Surgeons	3	0

### Recommendation Reversals

There were 11 recommendation reversals (Table 2) from 10 guidelines (4–6,17–33). There were two reversals from the American Association for the Study of Liver Diseases (AASLD), two from the American Society for Gastrointestinal Endoscopy (ASGE), and one each from the American College of Gastroenterology (ACG), American Society of Colon and Rectal Surgeons (ASCRS), British Society of Gastroenterology (BSG), European Association for the Study of the Liver (EASL), European Crohn’s and Colitis Organization (ECCO), European Society of Gastrointestinal Endoscopy (ESGE), and Japan Gastroenterological Endoscopy Society (JGES). Seven reversals were for medical therapies, two for procedures (e.g., endoscopy), one for lifestyle modifications, and one for a diagnostic modality. When considering subspecialties within gastrointestinal disease, four reversals were related to luminal gastroenterology, four related to liver disease, and one each for gastrointestinal oncology, inflammatory bowel disease, and pancreatic disease respectively (Table 2).

**Table 2.** Characteristics of recommendation reversals

Guideline	Reversal topic	Society	Type of practice	Area of gastroenterology
Murray and Carithers Jr. (2005) (18) Martin et al. (2013) (19)	Obesity in liver transplantation	AASLD	Lifestyle	Liver disease
Polson and Lee (2005) (20) Lee et al. (2011) (21)	Pulmonary artery catheterization for volume assessment in acute liver failure	AASLD	Diagnostic	Liver disease
O’Shea et al. (2010) (34) Singal et al. (2018) (5)	Pentoxifylline for alcoholic hepatitis	ACG	Medical	Liver disease
Steele et al. (2012) (23) Stewart et al. (2018) (24)	Radiation therapy for anal squamous cell cancer	ASCRS	Procedural	Gastrointestinal oncology
Hirota et al. (2003) (25) Banerjee et al. (2008) (26)	Antibiotic prophylaxis before endoscopy to prevent endocarditis	ASGE	Medical	Luminal gastroenterology
Hirota et al. (2003) (25) Banerjee et al. (2008) (26)	Antibiotic prophylaxis before endoscopy to prevent hardware, graft, and device infections	ASGE	Medical	Luminal gastroenterology
Jalan and Hayes (2000) (27) Tripathi et al. (2015) (4)	Isosorbide mononitrate for variceal bleeding prophylaxis	BSG	Medical	Luminal gastroenterology
European Association for the Study of Liver Diseases (2012) (28) Thursz et al. (2018) (29)	Pentoxifylline for alcoholic hepatitis	EASL	Medical	Liver disease
Dignas et al. (2010) (6) Gomollon et al. (2016) (8)	Thiopurine therapy in Crohn’s disease	ECCO	Medical	Inflammatory bowel disease
Dumonceau et al. (2012) (30) Dumonceau et al. (2018) (31)	Management of pancreatic stones	ESGE	Procedural	Pancreatic disease
Fujimoto et al. (2014) (32) Kato et al. (2018) (33)	Anticoagulation management before endoscopy	JGES	Medical	Luminal gastroenterology

AASLD, American Association for the Study of Liver Diseases; ACG, American College of Gastroenterology; ASCRS, American Society of Colon and Rectal Surgeons; ASGE, American Society for Gastrointestinal Endoscopy; BSG, British Society of Gastroenterology; ECCO, European Crohn’s and Colitis Organization; EASL, European Association for the Study of the Liver; ESGE, European Society of Gastrointestinal Endoscopy; JGES, Japan Gastroenterological Endoscopy Society.

### Recommendation Strength and Quality of Evidence

New evidence presented in guidelines for 10 recommendation reversals, with 1 reversal lacking new data (20,21). Meta-analyses were cited for two reversals, and randomized controlled trials (RCTs) for seven reversals (Table 3). The other evidence cited for reversals included single-arm trials, observational studies, narrative reviews, and other clinical guidelines. Recommendations were stronger after the reversal for three cases, weaker in two cases, and of similar strength in three cases (Table 4). We were unable to compare recommendation strengths for three reversals because one iteration did not provide a recommendation strength.

**Table 3.** Description of reversals and evidence base

Guidelines	Recommendation in first iteration	Recommendation in second iteration	Cited evidence in first iteration	Cited evidence in second iteration
Murray and Carithers Jr. (2005) (18) Martin et al. (2013) (19)	The 2005 AASLD guideline on liver transplantation listed morbid obesity (BMI >40 kg/m^2^) as a contraindication to liver transplantation.	The 2013 AASLD guideline on liver transplantation suggested that morbid obesity be only considered a relative contraindication, that patients attempt supervised weight loss, and that innovative approaches such as gastric sleeve operation simultaneous to transplantation be considered.	The original recommendation was based on evidence from a prospective cohort study suggesting an association between morbid obesity (>40 kg/m^2^) and decreased postoperative survival (35). 1 observational study	This reversal was based on evidence from an observational study. This prospective cohort study suggested that the severity of underlying liver disease in obese patients increased the risk of poor outcomes, rather than obesity in itself (36). 1 observational study
Polson and Lee (2005) (20) Lee et al. (2011) (21)	The 2005 AASLD guideline on acute liver failure suggested that pulmonary artery catheterization be considered in hemodynamically unstable patients with acute liver failure to ensure appropriate volume resuscitation.	The 2011 AASLD guideline on acute liver failure stated that pulmonary artery catheterization is rarely needed in acute liver failure and associated with significant morbidity.	There were no studies cited for the original recommendation.	There were no studies cited for this reversal.
O’Shea et al. (2010) (34) Singal et al. (2018) (5)	The 2010 ACG guideline on alcoholic hepatitis suggested considering pentoxifylline therapy for severe disease.	The 2018 ACG guideline on alcoholic hepatitis stated that pentoxifylline for severe alcoholic hepatitis is not supported by the evidence.	The original recommendation was supported by one randomized placebo- controlled clinical trial, which showed lower mortality in patients with severe alcoholic hepatitis treated with pentoxifylline compared to patients that received placebo (37). 1 RCT	This reversal was based on one meta-analysis of 10 studies showed no survival benefit and one meta-analysis of 22 studies that showed a short- term survival (38) benefit based on low-quality evidence (39). There were also nine RCTs which showed that pentoxifylline did not improve short-term survival compared to placebo (40–43),
				compared to corticosteroids (44,45), as an adjunct to corticosteroids (46,47), and among patients with no response to corticosteroids (48). 2 meta-analyses and 9 RCTs
Steele et al. (2012) (23) Stewart et al. (2018) (24)	The 2012 ASCRS guideline on anal squamous neoplasms recommended that higher doses of radiation therapy (56–60 GY for primary tumor and up to 65 GY for T3/4 lesions) without prolonged breaks when tolerated.	The 2018 ASCRS guideline on anal squamous neoplasms stated that no oncologic benefit exists for radiation doses >59 GY.	The original recommendation was based on a single-institution retrospective series (49) that showed improved locoregional control with higher doses of radiation therapy (56–60 GY for primary tumor and up to 65 GY for T3/4 lesions). Finally, a retrospective analysis of clinical trial data suggested that additional doses may increase the incidence of late morbidity (50). 2 observational studies	This reversal was based on a single-arm trial with a historical control in which patients with radiation doses of 59.6 GY had no improvement in locoregional control but higher colostomy rates compared to patients who received 40.0–50.4 GY^59^. Additionally, one RCT found no benefit to increasing radiation dosing using high-dose boost treatments (51). 1 single-arm trial
Hirota et al. (2003) (25) Banerjee et al. (2008) (26)	The 2003 ASGE guideline on antibiotic prophylaxis for endoscopic procedures recommended antibiotic prophylaxis to prevent endocarditis in high-risk patients undergoing procedures with increased rates of transient bacteremia.	The 2008 ASGE guideline on antibiotic prophylaxis for endoscopic procedures stated that aantibiotic prophylaxis is no longer recommended before endoscopy solely to prevent endocarditis.	The original recommendation was based on an AHA guideline (52). 1 guideline	This reversal was based on an AHA guideline (53). 1 guideline
Hirota et al. (2003) (25) Banerjee et al. (2008) (26)	The 2003 ASGE guideline on antibiotic prophylaxis for endoscopic procedures recommended antibiotic prophylaxis for patients with synthetic vascular grafts less than one year old.	The 2008 ASGE guideline on antibiotic prophylaxis for endoscopic procedures stated that antibiotic prophylaxis is no longer recommended before endoscopy for patients with vascular grafts.	The original recommendation was based on two experimental studies using animal models (54,55). 2 animal studies	This reversal was based on an AHA narrative review, which stated there was no evidence for infections associated with endoscopy in patients with any kind of nonvalvular cardiovascular devices (56). 1 narrative review
Jalan and Hayes (2000) (27) Tripathi et al. (2015) (4)	The 2000 BSG guideline on variceal hemorrhage recommended isosorbide mononitrate as first line treatment for primary prophylaxis when propranolol nor variceal band ligation could be used.	The 2015 BSG guideline on variceal hemorrhage recommended against using isosorbide mononitrate for primary prophylaxis	The original recommendation was based on two RCTs (57,58) which showed that there is no significant difference between isosorbide mononitrate and propranolol. 2 RCTs	This reversal was based on an RCT (59) which suggested that isosorbide mononitrate does not reduce variceal bleeding or mortality compared to placebo. 1 RCT
European Association for the Study of Liver Diseases (2012) (28) Thursz et al. (2018) (29)	The 2012 EASL guideline on alcoholic hepatitis recommended pentoxifylline as first line therapy in patients with severe disease and ongoing sepsis.	The 2018 EASL guideline on alcoholic hepatitis stated that pentoxifylline can no longer be recommended due to very weak evidence.	The original recommendation was based on three RCTs. One trial demonstrated that pentoxifylline reduced the incidence of hepatorenal syndrome without significant changes in liver function (37). Two other RCTs showed that pentoxifylline had a preventive effect on hepatorenal syndrome (40,45). 3 RCTs	This reversal was based on four RCTs which showed that pentoxifylline did not improve short-term survival compared to placebo (40), compared to corticosteroids (44), as an adjunct to corticosteroids (46), and among patients with no response to corticosteroids (48). 4 RCTs
Dignass et al. (2010) (6) Gomollon et al. (2016) (8)	The 2010 ECCO guideline on the management of Crohn’s disease stated that patients who have poor clinical prognostic factors are most suitable for early thiopurine, methotrexate and or anti-TNF therapy.	The 2016 ECCO guideline on the management of Crohn’s disease recommended against the early introduction of thiopurine therapy with newly diagnosed Crohn’s disease to maintain remission	The original recommendation was based on two RCTs suggested that early introduction of thiopurines with infliximab (60) or corticosteroids (61) can improve success rate. 2 RCTs	This reversal was based on one randomized trial in which patients did not have significantly different clinical remission rates but higher adverse event rates with azathioprine maintenance therapy compared to placebo (7). 1 RCT
Dumonceau et al. (2012) (30) Dumonceau et al. (2018) (31)	The 2012 ESGE guideline on chronic pancreatitis recommended ESWL as a first step, followed immediately by endoscopic extraction of stone fragments for radiopaque stones ≥5mm obstructing the main pancreatic duct.	The 2018 ESGE guideline on chronic pancreatitis suggested ESWL alone as first line for the clearance of radiopaque stones ≥5mm obstructing the main pancreatic duct, and only adding endoscopic therapy if there is no spontaneous clearance of stones.	The original recommendation did not cite evidence that directly support performing endoscopic extraction immediately after ESWL.	This reversal was based on one RCT (62) and one prospective cohort study (63) which demonstrated no benefit of ESWL with endoscopy compared to ESWL alone. 1 RCT and 1 observational study:
Fujimoto et al. (2014) (32) Kato et al. (2018) (33)	The 2014 JGES guideline on peri-endoscopic anticoagulant management suggests replacing warfarin or dabigatran with heparin for procedures with a high risk of bleeding.	The 2018 JGES guideline on peri-endoscopic anticoagulant management suggested continuing warfarin or using a direct oral anticoagulant (for non- valvular atrial fibrillation).	The original recommendation was based on a prospective cohort study (64) that investigated the pharmacokinetics of warfarin, a review article on the mechanism of action of heparin (65) and a prospective observational study which suggested replacing warfarin with heparin in patients at high risk of thromboembolism (66). 2 observational studies and 1 review	This reversal was based on two meta-analysis (67,68), one RCT (69), and two observational studies (70,71) which all suggested increased post-procedure bleeding risk with heparin. 2 meta-analyses, 1 RCT, and 2 observational studies:

AASLD, American Association for the Study of Liver Diseases; ACG, American College of Gastroenterology; ACC, American College of Cardiology; AHA, American Heart Association; ASGE, American Society for Gastrointestinal Endoscopy; BMI, body mass index; EASL, European Association for the Study of Liver Diseases; ECCO, European Crohn’s and Colitis Organization; ESGE, European Society of Gastrointestinal Endoscopy; ESWL, endoscopic shockwave lithotripsy; JGES, Japanese Gastroenterological Endoscopy Society; RCT, randomized controlled trial; TNF, tumor necrosis factor.

**Table 4.** Strength and level of evidence for recommendation reversals

Guideline	Reversal topic	Strength of evidence		Change in recommendation strength
		First iteration	Final iteration	
Murray and Carithers Jr. (2005) (18) Martin et al. (2013) (19)	Obesity in liver transplantation	USPTF recommendation (with no strength provided) based on level II-3 evidence (*multiple time series or dramatic uncontrolled experiments*).	GRADE weak recommendation (*trade- offs between desirable and undesirable effects are less certain*) based on moderate quality evidence (*further research is likely to have an important impact on confidence in the estimate of effect and may change the estimate*).	Similar recommendation strength between iterations
Polson and Lee (2005) (20) Lee et al. (2011) (21)	Pulmonary artery catheterization for volume assessment in acute liver failure	USPTF recommendation based on level III evidence (*expert opinion or descriptive epidemiology*).	USPTF recommendation based on level III evidence (*expert opinion or descriptive epidemiology*).	Similar recommendation strength between iterations
O’Shea et al. (2010) (34) Singal et al. (2018) (5)	Pentoxifylline for alcoholic hepatitis	AHA/ACC Class I recommendation (*conditions for which there is evidence and / or general agreement that a given diagnostic evaluation,* *procedure or treatment is beneficial, useful, and effective*) based on level B evidence (*derived from a single randomized trial or nonrandomized studies*).	GRADE conditional recommendation (*desirable effects probably outweigh the undesirable effects but uncertainty exists*) based on low quality evidence (*further research is very likely to have an important impact on our confidence in the estimate of effect and is likely to change the estimate*).	Weaker recommendation strength in final iteration
Steele et al. (2012) (23) Stewart et al. (2018) (24)	Radiation therapy for anal squamous cell cancer	GRADE weak recommendation (*trade-offs between desirable and undesirable effects are less certain*) based on moderate quality evidence (*RCTs with important limitations [inconsistent results, methodological flaws, indirect, or imprecise] or exceptionally strong evidence from observational studies*).	GRADE strong recommendation (*benefits clearly outweigh risk or burdens*) based on moderate quality evidence (*RCTs with important limitations [inconsistent results, methodological flaws, indirect, or imprecise] or exceptionally strong evidence from observational studies*).	Stronger recommendation strength in final iteration
Hirota et al. (2003) (25) Banerjee et al. (2008) (26)	Antibiotic prophylaxis before endoscopy to prevent endocarditis	Recommendation (with no strength provided) based on level C evidence (*expert opinion*).	GRADE strong recommendation (*benefits clearly outweigh risk or burden)* based on level C+ (*results from closely related RCTs that can be unequivocally extrapolated or overwhelming evidence from observational studies*).	Unable to compare as no recommendation strength provided in first iteration.
Hirota et al. (2003) (25) Banerjee et al. (2008) (26)	Antibiotic prophylaxis before endoscopy to prevent hardware, graft, and device infections	Recommendation (with no strength provided) based on level C evidence (*expert opinion*).	GRADE strong recommendation (*benefits clearly outweigh risk or burden)* based on level C+ (*results from closely related RCTs that can be unequivocally extrapolated or overwhelming evidence from observational studies*).	Unable to compare as no recommendation strength provided in first iteration.
Jalan and Hayes (2000) (27) Tripathi et al. (2015) (4)	Isosorbide mononitrate for variceal bleeding prophylaxis	NEEBG moderate recommendation (*rated as moderately important by the guideline panel*) based on strong evidence (*well designed RCTs, meta-analyses, or systematic reviews*).	OCEBM grade A recommendation (*consistent level 1 studies)* based on level 1b evidence (*individual RCT with narrow confidence interval*).	Stronger recommendation strength in final iteration
European Association for the Study of Liver Diseases (2012) (28) Thursz et al. (2018) (29)	Pentoxifylline for alcoholic hepatitis	GRADE strong recommendation (*benefits clearly outweigh risk or burden*) based on moderate quality evidence (*further research is likely to have an important impact on confidence in the estimate of effect and may change the estimate)*.	GRADE recommendation (with no strength provided) based on level 1 evidence (*data derived from meta-analyses or systematic reviews or from randomized trials with high quality*).	Unable to compare as no recommendation strength provided in final iteration.
Dignass et al. (2010) (6) Gomollon et al. (2016) (8)	Thiopurine therapy in Crohn’s disease	OCEBM grade D recommendation (*level 5 evidence or troublingly inconsistent or inconclusive* *studies of any level*) based level 5 evidence (*expert opinion without explicit critical appraisal, or based on physiology, bench research or “first principles”*).	GRADE weak recommendation (*trade-offs between desirable and undesirable effects are less certain*) based on low quality evidence.	Similar recommendation strength between iterations
Dumonceau et al. (2012) (30) Dumonceau et al. (2018) (31)	Management of pancreatic stones	SIGN grade B recommendation (*evidence including studies rated as 2++ directly applicable to the target population and demonstrating overall consistency of results or extrapolated evidence from studies rated as 1++ or 1+*) based on level 2+ evidence (*well conducted case–control or cohort studies with a low risk of confounding, bias, or chance, and a moderate probability that the relationship is causal*).	GRADE strong recommendation (*benefits clearly outweigh risk or burdens*) based on moderate quality evidence (*further research is likely to have an important impact on confidence in the estimate of effect and may change the estimate)*.	Stronger recommendation strength in final iteration
Fujimoto et al. (2014) (32) Kato et al. (2018) (33)	Anticoagulation management before endoscopy	MINDS level B recommendation (*reasonable scientific evidence available*) with no evidence level provided.	MINDS weak recommendation (*weakly recommended or suggested*) based on evidence level C (*weak evidence*).	Weaker recommendation strength in final iteration

GRADE, Grading of Recommendations Assessment, Development, and Evaluation; MINDS, Medical Information Network Distribution Service; NEEBG, North of England evidence based guidelines; OCEBM, Oxford Centre for Evidence Based Medicine; SIGN, Scottish Intercollegiate Guidelines Network; USPSTF, United States Preventive Services Task Force.

## Discussion

In this study of gastroenterology CPGs, we found 11 recommendation reversals from 1990 to 2019. These reversals were from major gastroenterology society guidelines and most commonly related to luminal gastroenterology and liver disease. Most reversals were based on evidence that identified harm or a lack of efficacy for established interventions. To our knowledge, this is the first study to evaluate reversals among gastroenterology CPG recommendations.

We propose to categorize the reversals in this study post-hoc into three groups. While we recognize that these categorizations are not mutually exclusive, they can provide a conceptual framework of how reversals can arise. In the first group, initial recommendations based on low quality evidence, such as expert opinion, were reversed in subsequent guidelines after higher quality studies were published. For example, the 2010 ECCO guideline on the management of Crohn’s disease suggested that patients who have poor clinical prognostic factors may be suitable for early thiopurine therapy based on expert opinion and physiological considerations (6). When a large randomized study was conducted however, patients receiving azathioprine experienced more harm compared to those who received placebo (7). As a result, the recommendation was reversed in 2016 (8). A similar pattern can be found for reversals related to radiation doses higher than 59Gy for anal squamous neoplasms (23,24), endoscopic shock wave lithotripsy combined with endoscopic retrograde cholangiopancreatography for pancreatic stones (30,31), and bridging anticoagulation for patients on warfarin before endoscopy (32,33). For two of the above cases, guideline authors assigned a higher recommendation strength (e.g., strong recommendation) after the reversal when supported by higher-quality data.

In the second group, recommendations based on high quality studies, such as randomized trials, were reversed after subsequent studies found conflicting results. For example, the 2010 ACG (34) and 2012 EASL (28) guidelines on alcoholic hepatitis recommended pentoxifylline in certain situations based on an RCT showing a 30-day mortality benefit with pentoxifylline compared to placebo (37). There were however, nine further RCTs which showed that pentoxifylline did not improve short-term survival compared to placebo (40–43), compared to corticosteroids (44,45), as an adjunct to corticosteroids (46,47), and among patients with no response to corticosteroids (48). As a result, the 2018 ACG (5) and EASL (29) guidelines recommended against using pentoxifylline in alcoholic hepatitis. Similarly, a recommendation for isosorbide mononitrate as primary prophylaxis of variceal hemorrhage (27), based on an RCT demonstrating similar efficacy to nadolol (57), was reversed (4) after a larger trial showed its lack of efficacy compared to placebo (59). These cases highlight the importance of replication even for seminal trials that guide practice. Estimates of efficacy of medical practices from single, small RCTs may be erroneous in further testing (72). Additionally, changes in the treatment and standards of care for specific diseases may alter the efficacy of earlier interventions (73).

In the third group, recommendations were reversed based on changes to practice in other medical specialties. A 2003 ASGE guideline recommended antibiotic prophylaxis before endoscopy for patients at high risk of developing endocarditis or with synthetic vascular grafts less than 1 year old (25). The 2008 ASGE guideline recommended against antibiotic therapy before endoscopy for both of the above circumstances (26), based American Heart Association documents (53,56). Similarly, a recommendation for pulmonary artery catheterization for assessment of shock in acute liver failure in a 2005 AASLD guideline (20) was reversed in a 2011 AASLD (21) guideline. This reversal likely reflected a practice change in critical care, where pulmonary artery catheterization was shown to have little benefit and substantial morbidity (14–16).

This study has several limitations. First, we may have missed reversals in guidelines published by societies that we did not include. Second, our small sample size of reversals precluded the ability to perform quantitative analysis. Third, there are not enough data in this study to comment on whether weak recommendations based on lower quality evidence are more likely to be reversed compared to recommendations supported by higher quality evidence. Fourth, while we stated whether recommendation reversals were made based on stronger or weaker evidence compared to the original recommendation, we did not analyze whether the initial recommendation was made prematurely. Fifth, we did not evaluate the quality of independent trials independently, but rather relied on the level and strength of evidence as judged by the guidelines included in this study. Finally, as there is no previously established definition of what constitutes a recommendation reversal, applying different definitions may lead other investigators to identifying other reversals.

Our findings reveal that recommendation reversals are infrequent in gastroenterology CPGs, though they may have important clinical consequences. Although prior research has found that 40% of studies in high-impact general medical journals find existing practices to be inferior to a prior standard (12,13,74), this result occurs among the subset of trials that rigorously test standard of care. For example, bispectral index monitoring, used in half of operating rooms in the United States by 2007 to detect anaesthesia awareness, was found to be no different to standardized sedation monitoring when tested in a large randomized study in 2008. Reversals in guidelines are much less frequent for several possible reasons. First, most gastroenterology guideline recommendations are based on low-quality evidence (9–11) such as expert opinion or physiologic principles. Testing such recommendations with robust, well controlled studies may occur rarely. Additionally, recommendations that are shown to be futile or harmful may persist in guidelines because subspecialty societies do not readily accept newer evidence contradicting their existing practices. A recent article found that compared to practices recommended by editorials in peer review journals, CPGs written by specialist societies were more likely to continue recommending those same practices despite new evidence (75). In gastroenterology, well controlled studies are needed to test practices rooted in poor evidence, and identify those that should be abandoned.

## CONCLUSION

In this large analysis of CPGs in gastroenterology, we identify only 11 recommendations that were subsequently reversed. Guideline reversals are rare, but pertain to important clinical questions and decisions. Future trials should seek to answer longstanding questions in gastroenterology and guideline committees should review post-hoc when new evidence comes to light. In order to deliver the best medical care to our patients, revising recommendations in CPGs based on new evidence is paramount.

## Supplementary Material

gwab040_suppl_Supplementary_Material
