# Supplementary material for: Recommendation Reversals in Gastroenterology Clinical Practice Guidelines
Source: J Can Assoc Gastroenterol. 2021 Oct 21;5(2):98–9. doi: 10.1093/jcag/gwab040 (PMC8972276; doi:10.1093/jcag/gwab040)
Supplement: gwab040_suppl_Supplementary_Material [file gwab040_suppl_supplementary_material.docx]

**Supplementary Table 1.** The twenty gastroenterology societies included in our search.

| AASLD (American Association of for the Study of Liver Diseases) |
| --- |
| ACG (American College of Gastroenterology) |
| AGA (American Gastroenterological Association) |
| ASCRS (American Society of Colon and Rectal Surgeons) |
| ASGE (American Society for Gastrointestinal Endoscopy) |
| ASPEN (American Society for Parenteral and Enteral Nutrition) |
| BSG (British Society of Gastroenterology) |
| CAG (Canadian Association of Gastroenterology) |
| EASL (European Association for the Study of the Liver) |
| ECCO (European Crohn’s and Colitis Organisation) |
| ESDO (European Society of Digestive Oncology) |
| ESGE (European Society of Gastrointestinal Endoscopy) |
| ESPEN (European Society for Clinical Nutrition and Metabolism) |
| GESA (Gastroenterological Society of Australia) |
| INASL (Indian National Association for Study of the Liver) |
| JGES (Japan Gastroenterological Endoscopy Society) |
| SAGES (Society of American Gastrointestinal and Endoscopic Surgeons) |
| UEG (United European Gastroenterology) |
| WEO (World Endoscopy Organization) |
| WGO (World Gastroenterology Organisation) |
